# Control of the Geometric Phase in Two Open Qubit–Cavity Systems Linked by a Waveguide

**DOI:** 10.3390/e22010085

**Published:** 2020-01-10

**Authors:** Abdel-Baset A. Mohamed, Ibtisam Masmali

**Affiliations:** 1 Department of Mathematics, College of Science and Humanities, Prince Sattam bin Abdulaziz University, Al-Aflaj 710-11912, Saudi Arabia; a.mohamed@psau.edu.sa; 2 Faculty of Science, Assiut University, Assiut 71516, Egypt; 3 Department of Mathematics, Faculty of Science, Jazan University, Gizan 82785, Saudi Arabia

**Keywords:** geometric phase, cavity damping, optical fiber

## Abstract

We explore the geometric phase in a system of two non-interacting qubits embedded in two separated open cavities linked via an optical fiber and leaking photons to the external environment. The dynamical behavior of the generated geometric phase is investigated under the physical parameter effects of the coupling constants of both the qubit–cavity and the fiber–cavity interactions, the resonance/off-resonance qubit–field interactions, and the cavity dissipations. It is found that these the physical parameters lead to generating, disappearing and controlling the number and the shape (instantaneous/rectangular) of the geometric phase oscillations.

## 1. Introduction

The mathematical manipulations of the open quantum systems, of the qubit–field interactions, depend on the ability of solving the master-damping [1] and intrinsic-decoherence [2] equations, analytically/numerically. To remedy the problems of these manipulations, the quantum phenomena of the open systems were studied for limited physical circumstances [3,4,5,6,7].

The quantum geometric phase is a basic intrinsic feature in quantum mechanics that is used as the basis of quantum computation [8]. The evolution of quantum systems (from an initial wave function to final time-dependent wave function) is cyclic, if the final time-dependent wave function returns to its initial wave function. When the evolution of these quantum systems is not cyclic, the geometric phase no longer exhibits robustness and the pertinent quantity of interest is the total phase, that is called the Pancharatnam geometric phase (PGP) [9]. The PGP means physically that the initial and the final states interfere, and the amplitude of the inner product reflects the phase difference between the states. The PGP was performed experimentally in neutron interferometry [10,11].

After that, the geometric phase was defined explicitly by Berry [12] in adiabatic systems, it extended to the quantum states of nonadiabatic cyclic [13] and noncyclic [14,15] evolutions. Geometric phase was proposed to realize the geometric quantum computations for different quantum model as: ion traps [16], atoms in cavity field [17], and superconducting circuits [18]. The time-dependent geometric was investigated in more physical models as: the model of a cavity QED was filled with a nonlinear medium and containing a quantum well [19], the model of a phase qubit dispersively coupled to a lossy LC circuit [20] and the model of a trapped ion with Stark shift [21].

The physical models which describe the transmitting quantum state between qubits located in isolated cavities, which are linked by an optical fiber mode, are effective systems for constructing quantum networks. There are essential developments in using optical fibers for quantum communication on the single photon level [22]. These models are very important to design the quantum network [23,24]. The models of the isolated qubit–cavity systems have other applications as the realization of quantum gates [25,26].

On the other hand, the transmitting quantum in the models of the isolated open cavities has problems due to the interaction of the leaky cavities with the external environment. When these isolated qubit–cavity systems interact with the environment, the quantum computations are confronted to the loss of their coherence [24]. A way to get around this problem is to introduce geometric phase shifts [27]. The geometric phase of the open quantum systems are inevitably affected by the decoherence of the external environment [19].

In this work, the physical model and its differential equations are introduced in Section 2. The Pancharatnam geometric phase and its computational results for different initial wave functions will be presented in Section 3. In Section 4, we end up by a conclusion.

## 2. The Physical Model and Its Differential Equations

The model consists of two cavities, *A* and *B*, linked by a waveguide mode of an optical fiber mode, each cavity field interacting with a two-level system (qubits). The general Hamiltonian is given by

(1) $$\begin{array}{ccc} \hat{H} & = & \sum_{i=A,B}\omega^{i}{\hat{a}}_{i}^{†}{\hat{a}}_{i}+\frac{1}{2}\omega_{0}^{i}\sigma_{i}^{z}+\omega_{f}^{i}{\hat{f}}_{i}^{†}{\hat{f}}_{i}\\ & & \;\;+\sum_{i=A,B}\chi_{i}\left({\hat{a}}_{i}^{†}\sigma_{i}^{-}+{\hat{a}}_{i}\sigma_{i}^{+}\right)+\nu_{i}\left({\hat{f}}_{i}{\hat{a}}_{i}^{†}+{\hat{f}}_{i}^{†}{\hat{a}}_{i}\right), \end{array}$$

where the three terms represent the free Hamiltonian of the cavity modes, the qubits, and the fiber modes, respectively, while the last two terms represent the interactions between the qubits and the cavity modes, and between the fibers and the cavity modes, respectively. ${\hat{a}}_{i}^{†}$ and ${\hat{a}}_{i}$ represent the creation and annihilation operators of the *i*-th cavity field, whereas ${\hat{f}}_{i}$ represent the lower operator of the fiber modes. The $\omega^{i}$, $\omega_{0}^{i}$ and $\omega_{f}^{i}$ are the frequencies of the cavity modes, the qubits and the fiber modes, respectively. The $\sigma_{i}^{z}$ and $\sigma_{i}^{\pm }$ are the operators of the inversion Pauli’s spin and up and down matrices of the *i*-th qubit. The $\chi_{i}$ and $\nu_{i}$ designate respectively the coupling constants of the *i*-th qubit–field and the fiber–field interactions, which are real values, thus the Hamiltonian is hermitian.

Here, we consider the short fiber limit that applies in most realistic experimental situations [28,29,30,31]. In short fiber limit requires that $\frac{2l\bar{\nu }}{2\pi c}\ll 1$, where *l* is the length of fiber, *c* is the light velocity in fiber and $\bar{\nu }$ is the decay rate of the cavity fields into a continuum of fiber modes, only one resonant mode $\hat{f}$ of the fiber interacts with the cavity modes. In this case, $\omega_{i}=\omega_{f}^{i}=\omega$ and $\nu_{i}=\chi_{f}$. Therefore, the interaction picture of the total Hamiltonian of the atom–cavity–fiber combined system is

(2) $$\begin{array}{ccc} {\hat{H}}_{int} & = & \sum_{i=A,B}\{\Delta_{i}\sigma_{i}^{z}+\chi_{i}\left({\hat{a}}_{i}^{†}\sigma_{i}^{-}+{\hat{a}}_{i}\sigma_{i}^{+}\right)+\chi_{f}\left(\hat{f}{\hat{a}}_{i}^{†}+{\hat{f}}^{†}{\hat{a}}_{i}\right)\}. \end{array}$$

where, $\Delta_{i}=\frac{\delta_{i}}{2}$ and $\delta_{i}=\omega_{0}^{i}-\omega$ that represents the detuning between the *i*-th qubits and the fields that describes the resonance/off-resonance cases.

However, a real quantum system will unavoidably interact with its surrounding environment, and the dissipation will cause degradation of the non-classical effects. To study the dissipation effect on the geometric phase, the time evolution of the system, described by the density matrix $\hat{\rho }$, is governed by the master equation [32],

(3) $$\begin{array}{ccc} \frac{\partial \hat{\rho }}{\partial t} & = & -\left[\hat{H},\hat{\rho }\right]+\sum_{i=A,B}\kappa_{i}\left(2{\hat{a}}_{i}\rho {\hat{a}}_{i}^{†}-{\hat{a}}_{i}^{†}{\hat{a}}_{i}\rho -\rho {\hat{a}}_{i}^{†}{\hat{a}}_{i}\right). \end{array}$$

where $\kappa_{i}$ are the cavity dissipation constants.

If we consider a situation where the evolution preserving the total number of excitation inside the cavity (i.e., no photons are emitted from the cavities), then diagonal terms in the Lindblad generator are only considered and presented full density operator when no quanta being lost in the interval 0 to *t*. The excitation is sufficiently small so that at few correlation times, the quantum state just undergoes evolution while the probability of cavity loss is negligible, and the purity of the state is preserved. Therefore, in this case, the non-diagonal terms of the Lindblad generator $2{\hat{a}}_{i}\rho {\hat{a}}_{i}^{†}$ (which describes the escape of the cavity photons) can be neglected in Equation (3) and it becomes [32,33,34]

(4) $$i\frac{d}{dt}\hat{\rho }={\hat{H}}_{eff}\hat{\rho }-{\left(\rho {\hat{H}}_{eff}\right)}^{†},$$

where the $H_{eff}$ is non-Hermitian operator and is given by

(5) $$\begin{array}{ccc} H_{eff} & = & \hat{H}-i\kappa_{A}{\hat{a}}_{A}^{†}{\hat{a}}_{A}-i\kappa_{B}{\hat{a}}_{B}^{†}{\hat{a}}_{B}. \end{array}$$

The last imaginary terms means that the dissipation is added into the zero Green’s function or spectrum. By using Equation (5), the differential equation of the wave function is given by

(6) $$\frac{d}{dt}|\psi \left(t\right)\rangle =-i\;{\hat{H}}_{eff}\;|\psi \left(t\right)\rangle .$$

The model of Equation (2) describes the interactions between the *i*-qubits and *i*-cavity, and between the *i*-cavity field and the fiber field; therefore, the total number of the excitations is ${\hat{f}}^{†}\hat{f}+\sum_{i}\left({\hat{\sigma }}_{i}^{+}{\hat{\sigma }}_{i}^{-}+{\hat{a}}_{i}^{†}{\hat{a}}_{i}\right)$. Since the non-Hermitian Hamiltonian ${\hat{H}}_{eff}$ conserves the number of excitations in the system, we restrict the number of excitations to 3 and consider only single photon processes to contribute to the wave function of the total system. Therefore, in the two-qubit basis space: $\{|\varpi_{1}\rangle =|0_{A}0_{B}\rangle ,\;|\varpi_{2}\rangle =|0_{A}1_{B}\rangle ,|\varpi_{3}\rangle =|1_{A}0_{B}\rangle ,|\varpi_{4}\rangle =|1_{A}1_{B}\rangle \}$, the wave function of the system is given by

(7) $$\begin{array}{ccc} \;\;\;\;|\psi (t)\rangle & = & [\alpha_{1}|000\rangle +\alpha_{5}|001\rangle +\alpha_{9}|010\rangle +\alpha_{13}|011\rangle \\ & + & \alpha_{17}|100\rangle +\alpha_{21}|101\rangle +\alpha_{25}|110\rangle +\alpha_{29}\left|111\rangle ]\right|\varpi_{1}\rangle \\ & + & [\alpha_{2}|000\rangle +\alpha_{6}|001\rangle +\alpha_{10}|010\rangle +\alpha_{14}|011\rangle \\ & + & \alpha_{18}|100\rangle +\alpha_{22}|101\rangle +\alpha_{26}|110\rangle +\alpha_{30}\left|111\rangle ]\right|\varpi_{2}\rangle \\ & + & [\alpha_{3}|000\rangle +\alpha_{7}|001\rangle +\alpha_{11}|010\rangle +\alpha_{15}|011\rangle \\ & + & \alpha_{19}|100\rangle +\alpha_{23}|101\rangle +\alpha_{27}|110\rangle +\alpha_{31}\left|111\rangle ]\right|\varpi_{3}\rangle \\ & + & [\alpha_{4}|000\rangle +\alpha_{8}|001\rangle +\alpha_{12}|010\rangle +\alpha_{16}|011\rangle \\ & + & \alpha_{20}|100\rangle +\alpha_{24}|101\rangle +\alpha_{28}|110\rangle +\alpha_{32}\left|111\rangle ]\right|\varpi_{4}\rangle . \end{array}$$

where, the state |mnl〉 means that the *A*-cavity field in the state |m〉, the *B*-cavity field in the state |n〉 whereas the fiber state is |l〉.

The amplitudes $\alpha_{n}\left(n=1-32\right)$ are derived from Equation (6) and they verify the following differential equations:

(8) $$\begin{array}{ccc} {\dot{\alpha }}_{2} & = & -i\left(-\Delta_{A}+\Delta_{B}\right)\alpha_{2}-i\chi_{B}\alpha_{9},\;\\ {\dot{\alpha }}_{3} & = & -i\left(\Delta_{A}-\Delta_{B}\right)\alpha_{3}-i\chi_{A}\alpha_{17},\\ {\dot{\alpha }}_{4} & = & -i\left(\Delta_{A}+\Delta_{B}\right)\alpha_{4}-i\chi_{A}\alpha_{18}-i\chi_{B}\alpha_{11},\\ {\dot{\alpha }}_{5} & = & i\left(\Delta_{A}+\Delta_{B}\right)\alpha_{5}-i\chi_{f}\alpha_{9}-i\chi_{f}\alpha_{17},\\ {\dot{\alpha }}_{6} & = & -i\left(-\Delta_{A}+\Delta_{B}\right)\alpha_{6}-i\chi_{B}\alpha_{13}-i\chi_{f}\alpha_{10}-i\chi_{f}\alpha_{18},\\ {\dot{\alpha }}_{7} & = & -i\left(\Delta_{A}-\Delta_{B}\right)\alpha_{7}-i\chi_{A}\alpha_{21}-i\chi_{f}\alpha_{11}-i\chi_{f}\alpha_{19},\\ {\dot{\alpha }}_{8} & = & -i\left(\Delta_{A}+\Delta_{B}\right)\alpha_{8}-i\chi_{A}\alpha_{22}-i\chi_{f}\alpha_{15}-i\chi_{f}\alpha_{12}-i\chi_{f}\alpha_{20},\\ {\dot{\alpha }}_{9} & = & i\left(\Delta_{A}+\Delta_{B}\right)\alpha_{9}-i\chi_{B}\alpha_{2}-i\chi_{f}\alpha_{5}-\kappa_{B}\alpha_{9},\\ {\dot{\alpha }}_{10} & = & -i\left(-\Delta_{A}+\Delta_{B}\right)\alpha_{10}-i\chi_{f}\alpha_{6}-\kappa_{B}\alpha_{10},\\ {\dot{\alpha }}_{11} & = & -i\left(\Delta_{A}-\Delta_{B}\right)\alpha_{11}-i\chi_{A}\alpha_{25}-i\chi_{B}\alpha_{4}-i\chi_{f}\alpha_{7}-\kappa_{B}\alpha_{11},\\ {\dot{\alpha }}_{12} & = & -i\left(\Delta_{A}+\Delta_{B}\right)\alpha_{12}-i\chi_{A}\alpha_{26}-i\chi_{f}\alpha_{8}-\kappa_{B}\alpha_{12},\\ {\dot{\alpha }}_{13} & = & i\left(\Delta_{A}+\Delta_{B}\right)\alpha_{13}-i\chi_{B}\alpha_{6}-i\chi_{f}\alpha_{25}-\kappa_{B}\alpha_{13},\\ {\dot{\alpha }}_{14} & = & -i\left(-\Delta_{A}+\Delta_{B}\right)\alpha_{14}-i\chi_{f}\alpha_{26}-\kappa_{B}\alpha_{14},\\ {\dot{\alpha }}_{15} & = & -i\left(\Delta_{A}-\Delta_{B}\right)\alpha_{15}-i\chi_{A}\alpha_{29}-i\chi_{B}\alpha_{8}-i\chi_{f}\alpha_{27}-\kappa_{B}\alpha_{15},\\ {\dot{\alpha }}_{16} & = & -i\left(\Delta_{A}+\Delta_{B}\right)\alpha_{16}-i\chi_{A}\alpha_{30}-i\chi_{f}\alpha_{28}-\kappa_{B}\alpha_{16},\\ {\dot{\alpha }}_{17} & = & i\left(\Delta_{A}+\Delta_{B}\right)\alpha_{17}-i\chi_{A}\alpha_{3}-i\chi_{f}\alpha_{5}-\kappa_{A}\alpha_{17},\\ {\dot{\alpha }}_{18} & = & -i\left(-\Delta_{A}+\Delta_{B}\right)\alpha_{18}-i\chi_{A}\alpha_{4}-i\chi_{B}\alpha_{25}-i\chi_{f}\alpha_{6}-\kappa_{A}\alpha_{18},\\ {\dot{\alpha }}_{19} & = & -i\left(\Delta_{A}-\Delta_{B}\right)\alpha_{19}-i\chi_{f}\alpha_{7}-\kappa_{A}\alpha_{19}\\ {\dot{\alpha }}_{20} & = & -i\left(\Delta_{A}+\Delta_{B}\right)\alpha_{20}-i\chi_{B}\alpha_{27}-i\chi_{f}\alpha_{8}-\kappa_{A}\alpha_{20},\\ {\dot{\alpha }}_{21} & = & i\left(\Delta_{A}+\Delta_{B}\right)\alpha_{21}-i\chi_{A}\alpha_{7}-i\chi_{f}\alpha_{25}-\kappa_{A}\alpha_{21},\\ {\dot{\alpha }}_{22} & = & -i\left(-\Delta_{A}+\Delta_{B}\right)\alpha_{22}-i\chi_{A}\alpha_{8}-i\chi_{B}A_{29}-i\chi_{f}\alpha_{26}-\kappa_{A}\alpha_{22},\\ {\dot{\alpha }}_{23} & = & -i\left(\Delta_{A}-\Delta_{B}\right)\alpha_{23}-i\chi_{f}\alpha_{27}-\kappa_{A}\alpha_{23},\\ {\dot{\alpha }}_{24} & = & -i\left(\Delta_{A}+\Delta_{B}\right)\alpha_{24}-i\chi_{B}\alpha_{31}-i\chi_{f}\alpha_{28}-\kappa_{A}\alpha_{24},\\ {\dot{\alpha }}_{25} & = & i\left(\Delta_{A}+\Delta_{B}\right)\alpha_{25}-i\chi_{A}\alpha_{11}-i\chi_{B}\alpha_{18}-i\chi_{f}\alpha_{13}-i\chi_{f}\alpha_{21}-\left(\kappa_{A}+\kappa_{B}\right)\alpha_{25},\\ {\dot{\alpha }}_{26} & = & -i\left(-\Delta_{A}+\Delta_{B}\right)\alpha_{26}-i\chi_{A}\alpha_{12}-i\chi_{f}\alpha_{14}-i\chi_{f}\alpha_{22}-\left(\kappa_{A}+\kappa_{B}\right)\alpha_{26},\\ {\dot{\alpha }}_{27} & = & -i\left(\Delta_{A}-\Delta_{B}\right)\alpha_{27}-i\chi_{B}\alpha_{20}-i\chi_{f}\alpha_{15}-i\chi_{f}\alpha_{23}-\left(\kappa_{A}+\kappa_{B}\right)\alpha_{27},\\ {\dot{\alpha }}_{28} & = & -i\left(\Delta_{A}+\Delta_{B}\right)\alpha_{28}-i\chi_{f}\alpha_{16}-i\chi_{f}\alpha_{24}-\left(\kappa_{A}+\kappa_{B}\right)\alpha_{28},\\ {\dot{\alpha }}_{29} & = & i\left(\Delta_{A}+\Delta_{B}\right)\alpha_{29}-i\chi_{A}\alpha_{15}-i\chi_{B}\alpha_{22}-\left(\kappa_{A}+\kappa_{B}\right)\alpha_{29},\\ {\dot{\alpha }}_{30} & = & -i\left(-\Delta_{A}+\Delta_{B}\right)\alpha_{30}-i\chi_{A}\alpha_{16}-\left(\kappa_{A}+\kappa_{B}\right)\alpha_{30},\\ {\dot{\alpha }}_{31} & = & -i\left(\Delta_{A}-\Delta_{B}\right)\alpha_{31}-i\chi_{B}\alpha_{24}-\left(\kappa_{A}+\kappa_{B}\right)\alpha_{31}. \end{array}$$

Also,

$$\begin{array}{ccc} \alpha_{1}\left(t\right) & = & \alpha_{1}\left(0\right)e^{i(\Delta_{A}+\Delta_{B})t},\\ \alpha_{32}\left(t\right) & = & \alpha_{32}\left(0\right)e^{-i\left(\Delta_{A}+\Delta_{B}\right)t-\left(\kappa_{A}+\kappa_{B}\right)t}. \end{array}$$

To solve numerically the above differential equations in order to determine the wave function |ψ(t)〉, we assume that the total system is initially in two different maximally entangled states:

(9) $$\begin{array}{ccc} {\;\;\;\;|\psi \left(0\right)\rangle }_{1} & = & \frac{1}{\sqrt{5}}[(|001\rangle +|011\rangle +|101\rangle +|111\rangle )⊗|\varpi_{1}\rangle \\ & & \;+e^{i\varphi }\left|110\rangle ⊗\right|\varpi_{4}\rangle ]. \end{array}$$

(10) $$\begin{array}{ccc} {|\psi \left(0\right)\rangle }_{2} & = & \frac{1}{4}[|000\rangle +|001\rangle +|010\rangle +|011\rangle \\ & + & |100\rangle +|101\rangle +|110\rangle +\left|111\rangle \right](|\varpi_{2}\rangle +|\varpi_{3}\rangle ), \end{array}$$

where we take the phase angle $\varphi =\frac{\pi }{4}$. The type of entanglement of the initial states of Equations (9) and (10) is very useful for distributed quantum information processing [29,30,31], and it possible to realize them experimentally [35,36,37].

## 3. Geometric Phase and Its Computational Results

To perform computation using geometric phase, it is necessary to understand the relation between geometric phase and dissipation noise. In open systems, the dissipation leads to converting a system from a pure state to a mixed state that often describes via a density matrix, say ρ(t). For this case, Uhlmann mathematically extended the geometric phase to the case of non-unitary evolution of mixed states [38,39]. But, if the effective description of the open system is governed by the master equation that is derived by neglecting the non-diagonal terms, then the open system can be described by the nonHermitian Hamiltonian ${\hat{H}}_{eff}$. Therefore, the unitary evolution of the initial state |ψ(0)〉 is governed by the Schrödinger equation as

$$|\psi \left(0\right)\rangle \to |\psi \left(t\right)\rangle =\left(U\left(t\right)=e^{-i{\hat{H}}_{eff}t}\right)|\psi \left(0\right)\rangle .$$

In this case, the total geometric phase being the argument of 〈ψ(0)|ψ(t)〉. it is given by [9]

(11) $$G_{P}\left(t\right)=\arg\{\langle \psi \left(0\right)|e^{-i{\hat{H}}_{eff}t}\left|\psi \left(0\right)\rangle \right\}=\arg\left\{\langle \psi \left(0\right)|\psi \left(t\right)\rangle \right\},$$

that is Pancharatnam geometric phase (PGP). If ${\tilde{\alpha }}_{i}\left(0\right)$ are the amplitudes of complex conjugate transpose of the initial state, then the geometric phase has the following expression

(12) $$G_{P}\left(t\right)=\arg\left[\sum_{i=1}^{32}{\tilde{\alpha }}_{i}\left(0\right)\alpha_{i}\left(t\right)\right].$$

For the especial initial state $\frac{1}{\sqrt{2}}[|000\varpi_{1}\rangle +|111\varpi_{4}\rangle ]$ with $\kappa_{i}=0$, the exact expression of the geometric phase is given by

(13) $$\begin{array}{ccc} G_{P}\left(t\right) & = & \arg\left[\cos\left(\Delta_{A}+\Delta_{B}\right)t\right]=\left\{\begin{array}{cc} \pi , & \cos\theta <0;\\ 0, & \cos\theta >0. \end{array}\right. \end{array}$$

where $\theta =(\Delta_{A}+\Delta_{B})t$. With this the exact expression, we can measure the geometric phase analytically and verify the predictions of the numerical results.

In the numerical simulations, the geometric phase is investigated under the effects of all coupling constants which are in the units of megahertz (MHz), and accordingly the time *t* is in the units of microseconds (μs). This choice of units was suggested with experimental parameters [40,41].

### 3.1. Dynamics of GP of ${|\psi \left(0\right)\rangle }_{1}$

When the entire system is prepared initially in the state ${|\psi \left(0\right)\rangle }_{1}$, the dynamics of the GP are given by

(14) $$G_{P}\left(t\right)=\frac{1}{\sqrt{5}}\arg\left[\alpha_{5}\left(t\right)+\alpha_{13}\left(t\right)+\alpha_{21}\left(t\right)+e^{i\varphi }\alpha_{28}\left(t\right)+\alpha_{29}\left(t\right)\right]$$

**Figure 1:**
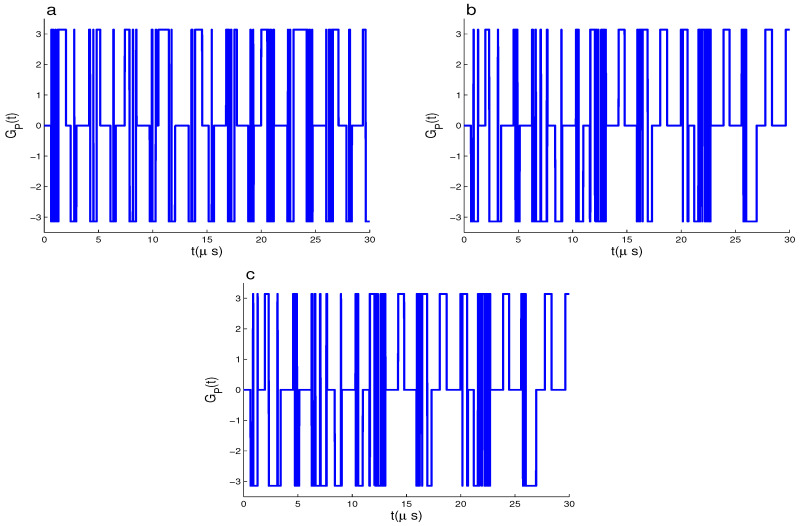
$G_{P}\left(t\right)$ for ${|\psi \left(0\right)\rangle }_{1}$ and $\delta_{i}=0$ with $\left(\chi_{A},\chi_{B},\chi_{f},\kappa \right)=\left(2,2,2,0\right)$ MHz in (**a**), $\left(\chi_{A},\chi_{B},\chi_{f},\kappa \right)=\left(0.5,2,2,0\right)$ MHz in (**b**), $\left(\chi_{A},\chi_{B},\chi_{f},\kappa \right)=\left(0.5,0.5,2,0\right)$ MHz in (**c**).

In Figure 1a, the geometric phase is plotted for the initial state ${|\psi \left(0\right)\rangle }_{1}$ and the strong coupling constants, $\left(\chi_{A},\chi_{B},\chi_{F}\right)=\left(2.0,2.0,2.0\right)$ MHz in the absence of the cavity dissipation effects, where $\kappa_{i}=\kappa =0\;\left(i=A,B\right)$. We note that; (1) The PGP arises from initial zero-value to oscillating between its extremes values, where the amplitudes of its oscillations satisfy the inequality $-\pi <G_{P}\left(t\right)<\pi$. The phenomena of the collapses and revivals appear as rectangular oscillations (it does not reach its extreme values instantly), where the PGP has invariant dynamics during some time intervals. (2) The coupling constants, $\left(\chi_{A},\chi_{B},\chi_{F}\right)=\left(2.0,2.0,2.0\right)$ MHz, lead to generating oscillations quickly during some intervals which are called *uncertain intervals* [19]. In these the intervals, the GP values can not be certainly determined.

Figure 1b–c shows that the dynamical behavior of the PGP is much sensitive to the coupling constants $\chi_{i}\left(i=A,B\right)$. If one of them is weakened (say $\chi_{A}=0.5$ MHz), the intervals of the collapse phenomenon ($G_{P}\left(t\right)=0$) increase. This observation is confirmed by weakening both the interaction couplings of the qubit–cavity systems, $\chi_{A}=\chi_{B}=0.5$ MHz.

**Figure 2:**
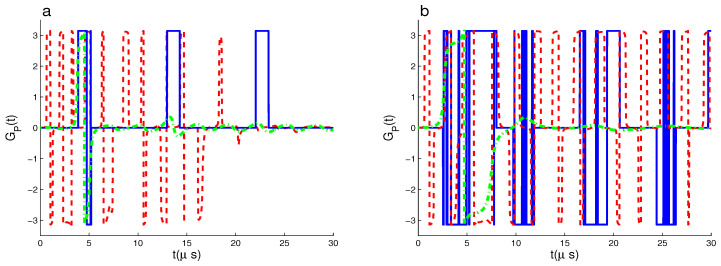
$G_{P}\left(t\right)$ for ${|\psi \left(0\right)\rangle }_{1}$ and $\delta_{i}=0$ with $\left(\chi_{A},\chi_{B},\chi_{f},\kappa \right)=\left(0.5,2,0.5,0.0\right)$ MHz (solid curve), $\left(\chi_{A},\chi_{B},\chi_{f},\kappa \right)=\left(0.5,2,2,0.2\right)$ MHz (dashed curve) and $\left(\chi_{A},\chi_{B},\chi_{f},\kappa \right)=\left(0.5,2,0.5,0.2\right)$ MHz (dashed-dotted curve) in (**a**). While in (**b**) for $\left(\chi_{A},\chi_{B},\chi_{f},\kappa \right)=\left(0.5,0.5,0.5,0.0\right)$ MHz (solid curves), $\left(\chi_{A},\chi_{B},\chi_{f},\kappa \right)=\left(0.5,0.5,2,0.2\right)$ MHz (dashed curve) and $\left(\chi_{A},\chi_{B},\chi_{f},\kappa \right)=\left(0.5,0.5,0.5,0.2\right)$ MHz (dashed-dotted curve).

The outcomes of the applying weak coupling constant between the fiber and the cavities are presented in the solid curves of Figure 2, where the dynamical behavior of $G_{P}\left(t\right)$ is displayed with ${|\psi \left(0\right)\rangle }_{1}$ and $\delta_{i}=0$ for $\left(\chi_{A},\chi_{B},\chi_{f},\kappa \right)=\left(0.5,2,0.5,0.0\right)$ MHz in (a), $\left(\chi_{A},\chi_{B},\chi_{f},\kappa \right)=\left(0.5,0.5,0.5,0.0\right)$ MHz in (b). We can observe that the oscillations of the PGP reduce by taking the small coupling constant $\chi_{f}=0.5$ MHz. The collapse intervals are during most of the chosen time interval. Whereas, if both the coupling constants $\chi_{B}$ and $\chi_{f}$ are wreaked, the collapse intervals decrease.

In general, with the large values of $\chi_{f}$, the generated PGP are more robust than that for small values. The effect strength of the small coupling constant $\chi_{f}$ on the number of the PGP oscillations and the appearance of the collapse/revival intervals depend on the coupling constants $\chi_{i}\left(i=A,B\right)$.

**Figure 3:**
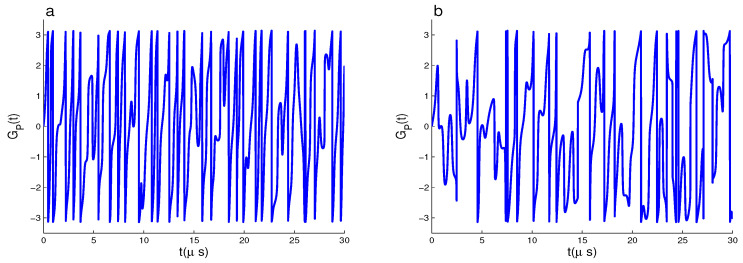
As Figure 1a but for $\delta_{A}=\delta_{B}=6$ MHz in (**a**) and $\left(\delta_{A},\delta_{B}\right)=\left(6,0\right)$ MHz in (**b**).

Dashed and dashed-dotted curves of the Figure 2 show the robustness of the PGP dynamical behavior against the dissipation coupling constants of the cavities $\kappa_{i}$ for $\left(\chi_{A},\chi_{B},\chi_{f},\kappa \right)=\left(0.5,2,2,0.2\right)$ MHz and $\left(\chi_{A},\chi_{B},\chi_{f},\kappa \right)=\left(0.5,2,0.5,0.2\right)$ MHz. We note that the cavity dissipation terms lead to: (1) The PGP has damped oscillatory dynamics, where the number and the amplitudes of its oscillations decrease clearly. After a particular time, the oscillatory behavior of $G_{P}\left(t\right)$ disappears and reaches its stationary zero-value approximately. (2) The damped oscillatory behavior of the PGP depends on the coupling constants of both the qubit–cavity and the fiber–cavity interactions. It disappears quickly with the cases $\left(\chi_{A},\chi_{B},\chi_{f},\kappa \right)=\left(0.5,0.5,2,0.2\right)$ MHz and $\left(\chi_{A},\chi_{B},\chi_{f},\kappa \right)=\left(0.5,0.5,0.5,0.2\right)$ MHz, see Figure 3b.

Figure 3 shows the effect of the detuning parameters $\delta_{i}$ on the dynamical behavior of the PGP. Where $G_{P}\left(t\right)$ is plotted as in Figure 1a, but for $\delta_{A}=\delta_{B}=6$ MHz in (a) and $\left(\delta_{A},\delta_{B}\right)=\left(6,0\right)$ MHz in (b). From Figure 3a, we not that the different off-resonance qubit–field interactions (non-zero detunings) of the $\delta_{i}=6$ MHz lead to: (1) The function $G_{P}\left(t\right)$ has more oscillations and it reaches its extreme values instantly. (2) The phenomena of the collapses and revivals, and the rectangular oscillations disappear completely. If the effects of both the off-resonance and resonance qubit–field interactions $\left(\delta_{A},\delta_{B}\right)=\left(6,0\right)$ MHz are combined, the number of the fluctuations of $G_{P}\left(t\right)$ are less these of the case $\delta_{i}=6$ MHz, see Figure 3b.

Finally, we can deduce that the robustness of the generated PGP depends on the coupling constants $\chi_{i}\;\left(i=A,B,f\right)$, the detuning parameters $\delta_{i}$ and the cavity dissipation κ.

### 3.2. Dynamics of GP of ${|\psi \left(0\right)\rangle }_{2}$

In this case, we will investigate the dependence of the generated PGP dynamics on the initial wave function of the total system. The PGP of the initial state ${|\psi \left(0\right)\rangle }_{2}$ is given by

(15) $$G_{P}\left(t\right)=\frac{1}{4}\arg\left[\sum_{k=0}^{7}\alpha_{2+4k}\left(t\right)+\alpha_{3+4k}\left(t\right)\right].$$

**Figure 4:**
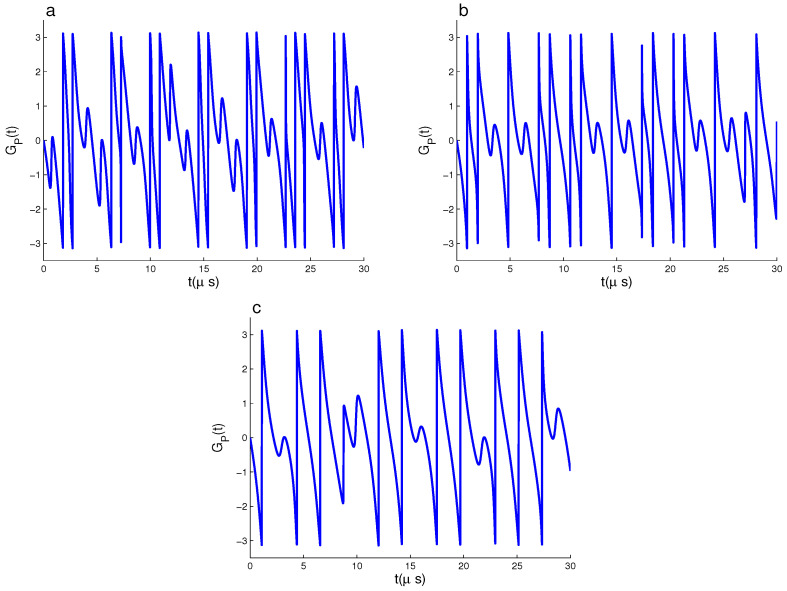
$G_{P}\left(t\right)$ for ${|\psi \left(0\right)\rangle }_{2}$ and $\delta_{i}=0$ with $\left(\chi_{A},\chi_{B},\chi_{f},\kappa \right)=\left(2,2,2,0\right)$ MHz in (**a**), $\left(\chi_{A},\chi_{B},\chi_{f},\kappa \right)=\left(0.5,2,2,0\right)$ MHz in (**b**), $\left(\chi_{A},\chi_{B},\chi_{f},\kappa \right)=\left(0.5,0.5,2,0\right)$ MHz in (**c**).

In Figure 4, the function $G_{P}\left(t\right)$ for the initial state ${|\psi \left(0\right)\rangle }_{2}$ is plotted for different sets of the coupling constants in the absence of the effects of both the cavity dissipations and the detuning parameters. By comparing the dynamical behaviors of PGP for ${|\psi \left(0\right)\rangle }_{1}$ of Figure 1, and for ${|\psi \left(0\right)\rangle }_{2}$ of Figure 4, we observe notable changes as: (1) The PGP has regular oscillatory behavior, where the $G_{P}\left(t\right)$ fluctuates instantly between its extreme values. The geometric phase of the initial state ${|\psi \left(0\right)\rangle }_{2}$ presents instantaneous oscillations unlike of ${|\psi \left(0\right)\rangle }_{1}$ that presents rectangular oscillations. (2) From Figure 4a–c, we find that the instantaneous oscillations may be reduced by weakening the coupling constants of the qubit–cavity systems.

**Figure 5:**
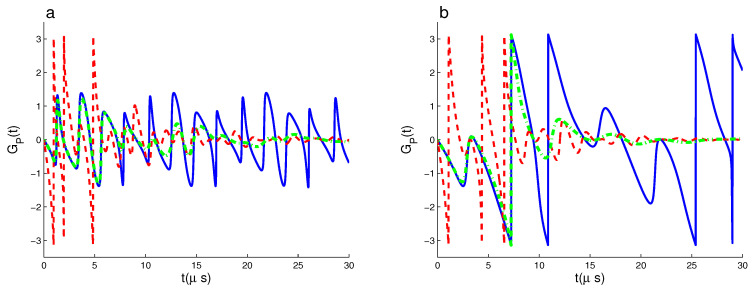
$G_{P}\left(t\right)$ for ${|\psi \left(0\right)\rangle }_{2}$ and $\delta_{i}=0$ with $\left(\chi_{A},\chi_{B},\chi_{f},\kappa \right)=\left(0.5,2,0.5,0.0\right)$ MHz (solid curve), $\left(\chi_{A},\chi_{B},\chi_{f},\kappa \right)=\left(0.5,2,2,0.2\right)$ MHz (dashed curve) and $\left(\chi_{A},\chi_{B},\chi_{f},\kappa \right)=\left(0.5,2,0.5,0.2\right)$ MHz (dashed-dotted curve) in (**a**). While in (**b**) for $\left(\chi_{A},\chi_{B},\chi_{f},\kappa \right)=\left(0.5,0.5,0.5,0.0\right)$ MHz (solid curves), $\left(\chi_{A},\chi_{B},\chi_{f},\kappa \right)=\left(0.5,0.5,2,0.2\right)$ MHz (dashed curve) and $\left(\chi_{A},\chi_{B},\chi_{f},\kappa \right)=\left(0.5,0.5,0.5,0.2\right)$ MHz (dashed-dotted curve).

Solid curves of Figure 5a,b show the dynamical behavior of the PGP for the initial state ${|\psi \left(0\right)\rangle }_{2}$ with the weak fiber–cavity interactions, $\chi_{f}=0.5$ MHz. We note that the amplitudes and the number of the instantaneous oscillations decrease with the small values of $\chi_{f}$.

Dashed and dashed-dotted curves of the Figure 5 show the robustness of the PGP dynamical behavior of the state ${|\psi \left(0\right)\rangle }_{2}$ against the cavity dissipations for different cases of $\left(\chi_{A},\chi_{B},\chi_{f},\kappa \right)$. With the large values of the cavity dissipation parameter, the instantaneous oscillations of PGP disappear quickly. Finally, we can deduce that the robustness of the generated PGP against the cavity dissipations depends on the chosen initial wave functions.

The predictions of the Pancharatnam phase are physically observable in more realistic experiments [42,43,44]. Where, the Pancharatnam phase is originally introduced to deal with the relative phase of two polarized light beams [9]. Therefore, the first experiment was tested the appearance of Pancharatnam’s phase in polarization states describing closed paths on the Poincaré sphere was performed by Bhandari and Samuel [42]. This test was however restricted to a limited set of two-level atom transformations. After that, alternative tests performed via employing unitary transformations, robust interferometric and polarimetric methods, and others [43,44]. Their experimental findings were in very good agreement with theoretical predictions [43].

## 4. Conclusions

Here, we consider two non-interacting two-level systems embedded in two separated open cavities linked via an optical fiber and leaking photons to the external environment. The geometric phase of the entire system is investigated numerically with two different chosen initial wave functions. It is found that, with the resonance qubit–field interactions and without the cavity dissipation effects, the strong coupling constants lead to generating the geometric phase with the collapse/revival phenomena and the rectangular oscillations. While, with the off-resonance qubit–field interactions, the geometric phase has more instantaneous oscillations without the collapse/revival phenomena, the rectangular oscillations disappear completely. If one or all of the coupling constants are weakened, these observations on the geometric phase have notable changes. It is found that the cavity dissipations lead to that the geometric phase has damped oscillatory behavior, and it reaches quakily its zero-value with the increase of the cavity dissipation parameter. The fast of the damped oscillatory dynamics can be controlled by the coupling constants. The physical models of two/more qubit–cavity systems linked by a waveguide mode have more potential applications in the generation of quantum correlations [24], the realization of quantum gates [25], distributed quantum computation [29], and quantum networking [45].
